# COVID-19 Vaccine Hesitancy and Acceptance Among Individuals With Cancer, Autoimmune Diseases, or Other Serious Comorbid Conditions: Cross-sectional, Internet-Based Survey

**DOI:** 10.2196/29872

**Published:** 2022-01-05

**Authors:** Richard Tsai, John Hervey, Kathleen Hoffman, Jessica Wood, Jennifer Johnson, Dana Deighton, Donald Clermont, Brian Loew, Stuart L Goldberg

**Affiliations:** 1 Inspire Arlington, VA United States; 2 Division of Outcomes Research John Theurer Cancer Center Hackensack Meridian School of Medicine Nutley, NJ United States

**Keywords:** COVID-19, vaccine, hesitancy, cancer, autoimmune diseases, vaccination, comorbidities, SARS-CoV-2, survey, cross-sectional, survey, incidence, safety, vulnerable, perception, attitude

## Abstract

**Background:**

Individuals with comorbid conditions have been disproportionately affected by COVID-19. Since regulatory trials of COVID-19 vaccines excluded those with immunocompromising conditions, few patients with cancer and autoimmune diseases were enrolled. With limited vaccine safety data available, vulnerable populations may have conflicted vaccine attitudes.

**Objective:**

We assessed the prevalence and independent predictors of COVID-19 vaccine hesitancy and acceptance among individuals with serious comorbidities and assessed self-reported side effects among those who had been vaccinated.

**Methods:**

We conducted a cross-sectional, 55-item, online survey, fielded January 15, 2021 through February 22, 2021, among a random sample of members of Inspire, an online health community of over 2.2 million individuals with comorbid conditions. Multivariable regression analysis was utilized to determine factors independently associated with vaccine hesitancy and acceptance.

**Results:**

Of the 996,500 members of the Inspire health community invited to participate, responses were received from 21,943 individuals (2.2%). Respondents resided in 123 countries (United States: 16,277/21,943, 74.2%), had a median age range of 56-65 years, were highly educated (college or postgraduate degree: 10,198/17,298, 58.9%), and had diverse political leanings. All respondents self-reported at least one comorbidity: cancer, 27.3% (5459/19,980); autoimmune diseases, 23.2% (4946/21,294); chronic lung diseases: 35.4% (7544/21,294). COVID-19 vaccine hesitancy was identified in 18.6% (3960/21,294), with 10.3% (2190/21,294) declaring that they would not, 3.5% (742/21,294) stating that they probably would not, and 4.8% (1028/21,294) not sure whether they would agree to be vaccinated. Hesitancy was expressed by the following patients: cancer, 13.4% (731/5459); autoimmune diseases, 19.4% (962/4947); chronic lung diseases: 17.8% (1344/7544). Positive predictors of vaccine acceptance included routine influenza vaccination (odds ratio [OR] 1.53), trust in responsible vaccine development (OR 14.04), residing in the United States (OR 1.31), and never smoked (OR 1.06). Hesitancy increased with a history of prior COVID-19 (OR 0.86), conservative political leaning (OR 0.93), younger age (OR 0.83), and lower education level (OR 0.90). One-quarter (5501/21,294, 25.8%) had received at least one COVID-19 vaccine injection, and 6.5% (1390/21,294) completed a 2-dose series. Following the first injection, 69.0% (3796/5501) self-reported local reactions, and 40.0% (2200/5501) self-reported systemic reactions, which increased following the second injection to 77.0% (1070/1390) and 67.0% (931/1390), respectively.

**Conclusions:**

In this survey of individuals with serious comorbid conditions, significant vaccine hesitancy remained. Assumptions that the most vulnerable would automatically accept COVID-19 vaccination are erroneous and thus call for health care team members to initiate discussions focusing on the impact of the vaccine on an individual’s underlying condition. Early self-reported side effect experiences among those who had already been vaccinated, as expressed by our population, should be reassuring and might be utilized to alleviate vaccine fears. Health care–related social media forums that rapidly disseminate accurate information about the COVID-19 vaccine may play an important role.

## Introduction

The rapid development of safe and effective vaccines against SARS-CoV-2 may stem the global COVID-19 pandemic. However, vaccine hesitancy—the reluctance or refusal to vaccinate—has emerged as a major worldwide public health concern, especially as it may impair the ability to reach herd immunity status [1-5]. An Ipsos poll of 15 countries for the World Economic Forum conducted in January 2021 reported vaccine acceptance rates ranging from 86% in Brazil to only 46% in Russia, with the United States ranking 12th (63% vaccine acceptance) [6]. Over time, COVID-19 vaccine acceptance has increased. Serial tracking polls by the Kaiser Family Foundation conducted in the United States reported that, as of July 2021, 70% of adults had either “received” or “will receive as soon as possible” the vaccine, up from 55% in February 2021 and 34% in early December 2020 [7,8]. However, antivaccination sentiment remained constant over this timeframe, with 14% stating that they would “definitely not get vaccinated” and 3% agreeing “only if required” [8]. Although the more virulent coronavirus Delta variant has increased the rapidity of vaccination uptake among individuals who were “waiting to see,” only 2% of those who refused vaccines were influenced by its emergence [8]. Multiple studies have explored reasons for COVID-19 vaccine hesitancy, with vaccine-specific concerns (side effects and efficacy), a need for more information, racial/ethnic biases, political views, general antivaccine attitudes or beliefs, and a lack of trust being most commonly cited [4,5,7-12].

Individuals with comorbid conditions have been disproportionately affected by COVID-19. A US review of nearly 500,000 commercially insured COVID-19 patients noted that, although only 51.7% had pre-existing conditions, 83.3% of the COVID-19–related deaths occurred among those with comorbidities. The risk of dying from COVID-19 was strongly correlated with the number of comorbidities, nearly doubling with a single comorbid condition and increasing 8-fold with 5 or more conditions [13]. Persons with developmental disorders, congenital and acquired neurologic disabilities, cancers (especially lung cancer, leukemia, and lymphoma), sickle cell disease, chronic kidney disease, heart failure, and diabetes appear to be at a particularly high risk for COVID-19–related mortality [13,14]. Hypertension, obesity, chronic lung diseases, and chronic liver diseases have also been associated with more severe COVID-19 disease [15-18].

COVID-19 vaccine allocation policies have prioritized individuals with serious comorbidities [19]. However, since regulatory clinical trials of COVID-19 vaccines excluded those with immunocompromising conditions and those receiving immunosuppressive therapies, few patients with cancer and autoimmune diseases were enrolled [20,21]. Thus, with limited vaccine safety and efficacy data available, but noting the increased mortality risk, patients with comorbidities may have conflicted COVID-19 vaccine attitudes. We therefore initiated an internet-based survey drawing from our international health-oriented social network to explore issues surrounding COVID-19 vaccine hesitancy in these vulnerable populations. Additionally, we sought to explore early self-reported side effect experiences among those who had already been vaccinated, as this might provide information useful to combating hesitancy.

## Methods

### Study Design and Participants

Survey participants were recruited from Inspire (Arlington, VA), an online health community of over 2.2 million individuals with comorbid conditions and their caregivers. Members anonymously engage with others with similar conditions through discussion posts and direct messaging. The community, with members residing in over 100 countries, represents over 3600 comorbid conditions including cancer, autoimmune diseases, rare diseases, and other chronic conditions.

When individuals join Inspire, they are given the opportunity of opting in to receive invitations for research projects. For this study, email invitations were sent on a daily basis to a computer-generated random sample of members who had agreed to receive research survey requests. Prior to participating in this study, individuals completed a consent form (approved by WCG IRB, Puyallup, WA) that detailed the purpose of the research. Participants were able to withdraw at any time throughout the survey. Participants were not compensated. Duplicate responses were removed by review of unique tokens assigned to participants.

### Measures

The survey consisted of 55 items, with initial responses leading to a potential addition of 8 follow-up questions. The survey was implemented using Alchemer, a web-based survey tool. Survey logic, programming, testing, and data validation were done via Alchemer. Items used to assess vaccine perception and hesitancy were adapted from Pew Research Center’s American Trends Panel 2020 survey, with additional questions added and linguistic adjustments [22]. Demographic, health conditions, and treatment-related questions were adapted from Inspire’s standard question sets. Behavioral and political leaning questions were adapted and modified from the Kaiser Family Foundation’s vaccine perception survey [7]. A dichotomous conservative political leaning variable was created from the multi-option political leaning question to include in the logistic regression analysis. This was done by grouping participants into either conservative political leaning or nonconservative political leaning categories.

Independent measures in the survey detailed demographics including age, education level, political leaning, ethnicity, income, residence (country of residence; if in the United States, state of residence), patient history of disease including specific disease, current treatment status if a cancer patient, and gender. Dependent measures included plans to receive the vaccine and attitudes and concerns toward the COVID-19 vaccines.

Interest in obtaining the vaccine was evaluated through the question, “Do you plan to get the COVID-19 vaccine when one is available?” This item was evaluated with options of “I already got it,” “I’ve tried but have not been able to get it,” “Definitely,” “Probably,” “Unsure,” “Probably not,” and “Definitely not.” For the purpose of analysis, participants who responded with “Definitely not,” “Probably not,” or “Unsure” were considered to be “vaccine hesitant.” Participants indicating the other responses, including those who had already received the COVID-19 vaccine, were considered to be “vaccine acceptant.”

Attitudes and concerns about the vaccine were elicited through the question, “What are your concerns about the vaccine? Check all that apply.” The possible responses included the following: “I do not believe I need it,” “I do not think it was developed responsibly,” “I do not trust the government has insured that the vaccines are safe and effective,” “I do not trust vaccines in general,” “I do not trust the COVID-19 vaccine in particular,” “I am concerned that the COVID-19 vaccine is too new,” “I want to see how others respond first,” “Concerns over the role of politics in the development process,” “It is too difficult to get vaccinated,” “I am concerned with contracting the coronavirus from the vaccine,” “I am concerned about the side effects or discomfort,” and “I have religious objections.” 

As concerns about side effects may contribute to COVID-19 vaccine hesitancy and since immunocompromised individuals were largely excluded from COVID-19 vaccine trials, we sought to obtain additional information about the experiences of individuals who had received the vaccine. Specifically, we included questions about the type of vaccine received and which (if any) side effects were experienced. The list of reportable symptoms and effects from the vaccine included on the survey were adapted from the Pfizer/BioNTech BNT162b2 mRNA COVID-19 Vaccine FDA Briefing Report [23]. Potential localized side effects included pain at the injection site, swelling at the injection site, redness at the injection site, itching at the injection site, and other. Potential systemic side effects included fever, chills, headache, joint pain, muscle/body aches, fatigue, nausea, vomiting, diarrhea, abdominal pain, rash, and other.

### Statistical Plan

Two-way cross tabulations were used to summarize sociodemographic variables, behavioral and public health belief variables, and comorbid disease variables across vaccine hesitancy. Pearson chi-squared tests were performed to assess for statistical significance in the differences between groups. Univariate logistic regression analyses were performed to assess independent relationships between several variables and the dichotomous vaccine acceptance variable.

Multivariate logistic regression analysis was performed to assess the relationship between multiple predictor variables and the dichotomized vaccine acceptance variable. Two-sided, design-based tests and an alpha level of .05 was used to evaluate statistical significance in all chi-squared, F test, and logistic regression analyses. No backward selection was used, and all variables remained in the model regardless of their significance level. All data management and analysis were conducted using SPSS Version 28 (IBM Corp, Armonk, NY).

### Study Funding

This study was funded by Inspire, which was responsible for the study design; the collection, analysis, and interpretation of the data; and the decision to approve publication of the finished manuscript.

## Results

### Survey Respondent Demographics

Invitations to participate in this survey were sent to 996,500 members of the Inspire health community between January 15, 2021 and February 22, 2021. Responses to the survey request were received from 21,943 individuals (2.2%), of which 17,115 completed the entire survey (1.7% of those invited and 78.0% of respondents). The median age range of respondents was 56-65 years, which appeared older than the Inspire community median age range of 40-49 years. The survey respondents were mostly female (15,696/20,685, 75.9%), similar to the general Inspire community (77%). There was minimal self-identification as belonging to a racial or ethnic minority within the respondent population.

Inspire’s membership is made up of both individuals with declared illnesses and their caregivers. However, caregivers who wished to participate in this study separate from their loved ones were instructed to complete a separate survey based on their own attitudes and to document their own health status. All participants (21,943/21,943, 100%) in this project indicated at least one comorbid condition. A cancer diagnosis was self-reported by 27.3% (5459/19,980) of responding participants, 23.2% (4946/21,294) had an autoimmune disease, and 35.4% (7544/21,294) were diagnosed with a chronic lung disease.

Respondents were highly educated, with 58.9% (10,198/17,298) holding college or postgraduate degrees. Political leanings were diverse, with 31.6% (5683/17,967) self-declaring liberal tendencies, 20.7% (3711/17,967) self-declaring as conservative, 24.3% (4357/17,967) self-declaring as independent, and 23.5% (4216/17,967) preferring not to declare. Respondents lived in 123 countries, with 74.2% (16,277/21,943) residing in the United States, 8.5% (1855/21,943) in Canada, 8.1% (1781/21,943) in the United Kingdom, 3.1% (688/21,943) in Australia, and the remaining 6.1% (1342/21,943) in Europe, Central, South America and the Caribbean, the Middle East, the Russian Federation, Africa, or the Far East.

### COVID-19 Vaccine Hesitancy in the Study Cohort

Among the 21,294 individuals with cancer, autoimmune diseases, or other serious diseases who responded to survey questions about their attitudes on vaccination, 18.6% (3960/21,294) indicated COVID-19 vaccine hesitancy, including 10.3% (2190/21,294) who declared that they would not receive the vaccine, 3.5% (742/21,294) who stated that they would probably not, and 4.8% (1028/21,294) who were not sure whether they would agree to be vaccinated. By contrast, 25.8% (5501/21,294) respondents reported that they had already received at least one COVID-19 vaccine injection by February 22, 2021. Of the US participants, 29.6% (4813/16,277) had already undergone vaccination. Among participants from other countries, 688 had undergone vaccination including 68% of participants living in Israel, 27% in the United Kingdom, 4% in Canada, and none in Australia. Additionally, 6.9% (1462/21,294) had tried but had been unable to obtain the vaccine, 43.9% (9342/21,294) definitely planned to undergo vaccination, and 4.8% (1029/21,294) indicated that they probably would undergo vaccination, leading to an overall vaccine acceptance of 81.4% (17,334/21,294).

### Factors Independently Associated With COVID-19 Vaccine Hesitancy

As shown in Table 1, multiple demographic factors were associated with vaccine hesitancy in the simple logistic regression analysis. Younger age was associated with increased vaccine hesitancy. In this survey of Inspire members with serious illnesses, 62.8% (12,707/20,225) of respondents were greater than 55 years of age, and in this subgroup, only 13.8% (1757/12,707) were vaccine hesitant compared with 25.1% (1889/7518) among those younger in age (*P*<.001). Although few self-reported a non-white racial or ethnic category, those who did report were more likely to be vaccine hesitant. The Inspire respondent members were highly educated, with 58.9% (10,198/17,298) possessing a college degree—a cohort who had vaccine hesitancy of 13.7% (1396/10,198) compared with 22.5% (1597/7100) among those with less formal education (*P*<.001). Respondents had diverse political leanings, but those with more conservative political leanings were more likely to express vaccine hesitancy. Respondents living outside the United States were more likely to be vaccine hesitant (998/4579, 21.8%) compared with those from the United States (2904/16,596, 17.5%; *P*<.001).

Opinions about public health policy also shaped vaccine hesitancy attitudes. In our study population of individuals with severe illnesses, 96.2% (18,376/19,468) reported routinely wearing masks. Although a greater proportion of mask wearers reported vaccine acceptance than those who reported not wearing masks, 18.4% (3444/18,736) of mask wearers remained vaccine hesitant. Most (16,269/21,294, 78.2%) respondents routinely received an influenza vaccination—a cohort with a vaccination acceptance prevalence of 91.6% (14,905/16,269) compared with the 45.9% (2083/4545) acceptance prevalence among those who did not routinely receive an influenza vaccine (*P*<.001). Respondents who did not feel that the media reported scientific data accurately had a slightly higher prevalence of vaccine hesitancy (635/3084, 20.6%) compared with those that did believe media information was scientifically accurate (1924/10,465, 18.4%; *P*=.006) Among those who responded “No” or “Probably not” to the question “Do you trust the vaccine was developed responsibly?”, 98.4% (1512/1537) and 91.0% (575/632), respectively, reported being vaccine hesitant (*P*<.001; Table 2).

**Table 1:** Vaccine hesitancy by age, gender, ethnicity, education level, and political leanings among individuals with serious comorbidities (n=21,294) using Inspire between January 15, 2021 and February 22, 2021.

Characteristic		Overall sample (n=21,294), n (%)	COVID-19 vaccine received or definitely or probably will receive the vaccine (n=17,334), n (%)	Definitely or probably will not receive the vaccine or unsure about receiving the vaccine (n=3960), n (%)
**Age^a^(years)**				
	<26	381 (1.9)	289 (75.9)	92 (24.1)
	26-35	1315 (6.5)	928 (70.6)	387 (29.4)
	36-45	2513 (12.4)	1871 (74.5)	642 (25.5)
	46-55	3309 (16.4)	2541 (76.8)	768 (23.2)
	56-65	5288 (26.2)	4340 (81.1)	948 (17.9)
	66-75	5591 (27.6)	4961 (88.7)	630 (11.3)
	>75	1828 (9.0)	1649 (90.2)	179 (9.8)
**Gender^b^**				
	Male	4989 (24.1)	4237 (84.6)	752 (15.4)
	Female	15,696 (75.9)	12,802 (81.5)	2894 (18.5)
**Race/ethnicity^c^**				
	White	17,354 (89.2)	14,487 (83.5)	2867 (16.5)
	Black or African American	514 (2.6)	391 (76.1)	123 (23.9)
	Hispanic or Latino	614 (3.2)	509 (82.9)	105 (17.1)
	Asian	627 (3.2)	520 (82.9)	107 (17.1)
	Hawaiian/Pacific Islander	22 (0.1)	15 (67.2)	7 (31.8)
	Native American/Alaskan	132 (0.7)	88 (66.7)	44 (33.3)
	Other	479 (2.5)	337 (70.4)	142 (29.6)
	Prefer not to answer	706 (3.6)	306 (43.4)	400 (56.6)
**Education level^d^**				
	High school or less	1640 (9.5)	1246 (75.9)	394 (24.1)
	Vocational or associate degree	2546 (14.7)	1955 (76.8)	591 (23.2)
	Some college	2914 (16.8)	2302 (79.0)	612 (21.0)
	College degree	4448 (25.7)	3748 (84.3)	700 (15.7)
	Postgraduate	5750 (33.2)	5054 (87.9)	696 (12.1)
**Political leaning^e^**				
	Liberal	5683 (31.6)	5401 (95.0)	282 (5.0)
	Conservative	3711 (20.7)	2653 (71.5)	1058 (28.5)
	Independent	4357 (24.3)	3520 (80.8)	837 (19.2)
	Prefer not to answer	4216 (23.5)	3185 (75.5)	1031 (24.5)

^a^n=20,225.

^b^n=20,685.

^c^n=19,465.

^d^n=17,298.

^e^n=17,967.

**Table 2:** Responses to the question, “Do you plan to get the COVID-19 vaccine when one is available?”, as an indicator of vaccine hesitancy, by mask wearing, routine influenza vaccination, belief in media coverage accuracy, and trust in responsible development among individuals with serious comorbidities (n=21,294) using Inspire between January 15, 2021 and February 22, 2021.

Characteristic		Overall sample (n=21,294), n (%)	Responses	
			“I already got it,” “I’ve tried but have not been able to get it,” “Definitely,” “Probably”, n (%)	“Unsure,” “Probably not,” “Definitely not”, n (%)
**Mask wearing^a^**				
	Always/sometimes wears a mask	18,736 (96.2)	15,292 (81.6)	3444 (18.4)
	Rarely/never wears a mask	732 (3.8)	557 (76.1)	175 (23.9)
**Routine influenza vaccine^b^**				
	Usually gets a flu vaccine	16,269 (78.2)	14,905 (91.6)	1364 (8.4)
	No flu vaccine	4545 (21.8)	2083 (45.9)	2462 (54.1)
**Media information scientifically accurate^c^**				
	Yes or generally yes	10,465 (53.8)	8541 (81.6)	1924 (18.4)
	No or generally no	3084 (15.8)	2449 (79.4)	635 (20.6)
	Mixed	5910 (30.3)	4852 (82.1)	1058 (17.9)
**Do you trust the vaccine was developed responsibly^d^**				
	Yes	12,498 (61.2)	12,292 (98.4)	206 (1.6)
	Probably so	3900 (19.1)	3494 (89.6)	406 (10.4)
	Not sure	1837 (9.0)	750 (40.8)	1087 (59.2)
	Probably not	632 (3.1)	57 (9.0)	575 (91.0)
	No	1537 (7.5)	25 (1.6)	1512 (98.4)

^a^n=19,468.

^b^n=20,814.

^c^n=19,459.

^d^n=20,409.

Of the survey respondents, 9.0% (1906/21,294) self-reported a prior history of COVID-19 infection, and an additional 5.1% (1085/21,294) believed that they had experienced symptoms suggestive of COVID-19 without confirmation (or were unsure). Although current guidelines recommend vaccination despite prior infection, 34.7% (1039/2991) of these individuals were vaccine hesitant. By contrast, among the more than 17,000 respondents who claimed no prior exposure to SARS-CoV-2, only 15.8% (2758/17,460) were vaccine hesitant (*P*<.001; Table 3).

**Table 3:** Responses to the question, “Do you plan to get the COVID-19 vaccine when one is available?”, as an indicator of vaccine hesitancy, among individuals with serious comorbidities (n=21,294) who used Inspire between January 15, 2021 and February 22, 2021, according to prior COVID-19 infection history (n=20,451).

Previous COVID-19 infection status	Overall sample (n=20,451), n (%)	Responses	
		“I already got it,” “I’ve tried but have not been able to get it,” “Definitely,” “Probably”, n (%)	“Unsure,” “Probably not,” “Definitely not”, n (%)
Had COVID-19	1906 (9.0)	1209 (63.4)	697 (36.6)
Unsure if had COVID-19	1085 (5.1)	743 (68.5)	342 (31.5)
Did not have COVID-19	17,460 (85.4)	14,702 (84.2)	2758 (15.8)

### Vaccine Hesitancy in Specific High-Risk Comorbid Populations

Among the 5459 individuals with cancer, 13.4% (731/5459) indicated vaccine hesitancy, including 13.2% (193/1463) of those who were currently receiving treatment and 13.5% (538/3996) of those who had completed prior treatment. Those who were not being treated for cancer had a vaccine hesitancy prevalence of 20.3% (2954/14,521). The difference in vaccine hesitancy proportions between those being treated for cancer and those not being treated for cancer was statistically significant (*P*<.001). Among participants with autoimmune diseases, 19.4% (962/4946) self-reported vaccine hesitancy compared with 18.0% (2943/16,348) of those not being treated for an autoimmune disease who reported vaccine hesitancy (*P*=.02). Of the respondents with chronic lung disease, 17.8% (1344/7544) reported vaccine hesitancy compared with 19.0% (2616/13,750) of those not being treated for chronic lung disease (*P*=.03). Vaccine hesitancy was also expressed by 19.7% (598/3041; *P*=.03) of those diagnosed as obese, 18.0% (963/5358; *P*=.99) diagnosed with hypertension, and 19.0% (266/1400; *P*=.30) of individuals living with type 2 diabetes, with comparisons against respondents who did not indicate these comorbidities (Table 4).

**Table 4:** Responses to the question, “Do you plan to get the COVID-19 vaccine when one is available?”, as an indicator of vaccine hesitancy, among individuals with serious comorbidities (n=21,294) using Inspire between January 15, 2021 and February 22, 2021.

Characteristic			Overall sample (n=21,294), n (%)		Responses		
					“I already got it,” “I’ve tried but have not been able to get it,” “Definitely,” “Probably”, n (%)		“Unsure,” “Probably not,” “Definitely not”, n (%)
**Cancer^a^**							
	Yes, in treatment	1463 (7.3)		1270 (88.8)		193 (13.2)	
	Yes, past treatment	3996 (20.0)		3458 (86.6)		538 (13.5)	
	No cancer	14,521 (72.7)		11,567 (79.7)		2954 (20.3)	
**Autoimmune disease**							
	Yes	4946 (23.2)		3984 (80.6)		962 (19.4)	
	No	16,348 (76.8)		13,405 (82.0)		2943 (18.0)	
**Chronic lung disease**							
	Yes	7544 (35.4)		6200 (82.2)		1344 (17.8)	
	No	13,750 (64.6)		11,134 (81.0)		2616 (19.0)	
**Hypertension**							
	Yes	5358 (25.2)		4395 (82.0)		963 (18.0)	
	No	15,936 (74.8)		13,068 (82.0)		2868 (18.0)	
**Type 2 diabetes**							
	Yes	1400 (6.6)		1134 (81.0)		266 (19.0)	
	No	19,894 (93.4)		16,353 (82.2)		3541 (17.8)	
**Obesity**							
	Yes	3041 (14.3)		2443 (80.3)		598 (19.7)	
	No	18,253 (85.7)		14,968 (82.0)		3285 (18.0)	

^a^n=19,980.

### Univariate Logistic Regression Analysis of COVID-19 Vaccine Acceptance

In the univariate logistic regression analysis, having received a routine influenza vaccine was associated with COVID-19 vaccine acceptance (odds ratio [OR] 1.24). Those who reported routinely receiving an influenza vaccine had 1.24 times the odds of being COVID-19 vaccine acceptant. Those who reported having trust that the COVID-19 vaccine was developed responsibly had 2.07 times the odds of being vaccine acceptant (OR 2.07). Those who reported being previously infected with COVID-19 had 0.93 times the odds of being vaccine hesitant (OR 0.93). Those who reported an independent political leaning or liberal political leaning had 1.12 and 1.14 times the odds, respectively, of being vaccine acceptant when compared with those who reported a conservative political leaning. Respondents residing within the United States had 1.03 times the odds of reporting vaccine acceptance than those living outside the United States. Those with an age higher than the median age of the study had 1.12 times the odds (or a 12% increase in odds) of reporting vaccine acceptance compared with those below the median age, while those at the median age had 0.99 times the odds of being vaccine acceptant compared with those below the median age. Moreover, those with some college education had 1.03 times the odds of being vaccine acceptant compared with those with a high school degree or less, while those with at least a 4-year degree had 1.04 times the odds of being vaccine acceptant compared with those with a high school degree or less. Smoking status was not significantly associated with vaccine acceptance. Men had 0.98 times the odds of being vaccine acceptant than women. Those diagnosed with cancer had 1.03 times the odds of being vaccine acceptant compared with those not diagnosed with cancer, and those who reported mask wearing had 1.02 times the odds of being vaccine acceptant (Table 5).

**Table 5:** Univariate logistic regression of vaccine acceptance among individuals with serious comorbidities (n=21,294) using Inspire between January 15, 2021 and February 22, 2021.

Variable			Odds ratio (95% CI)		*P* value
Routine influenza vaccine			1.24 (1.23-1.25)		<.001
Trust in responsible development of COVID vaccine			2.07 (2.05-2.09)		<.001
Prior COVID infection			0.93 (0.92-0.94)		<.001
**Political leaning**					
	Conservative political leaning (reference)	-^a^		-	
	Independent	1.12 (1.10-1.13)		.003	
	Liberal leaning	1.14 (1.12-1.15)		<.001	
Residence (United States vs outside the United States)			1.03 (1.02-1.04)		<.001
**Age**					
	Age below the median (reference)	-		-	
	Median age	0.99 (0.98-0.99)		<.001	
	Age above the median	1.12 (1.11-1.13)		<.001	
**Education level**					
	High school and less (reference)	-		-	
	Some college, associate degree	1.03 (1.03-1.04)		<.001	
	At least a college degree	1.04 (1.02-1.06)		<.001	
Smoking status			1.01 (1.00-1.02)		.17
Gender			0.98 (0.97-0.99)		.001
Cancer diagnosis			1.03 (1.02-1.04)		.001
Mask wearing			1.02 (1.01-1.03)		<.001

^a^Reference category.

### Multivariate Logistic Regression Models of COVID-19 Vaccine Acceptance

To understand the impact of these independent variables on vaccine acceptance, a multivariate logistic regression analysis was performed to predict those who had received or planned to receive their vaccination by February 20, 2021. Overall, our model was a statistically significant predictor of vaccine acceptance, with an adjusted *R^2^* of 0.525, meaning our model explained 52.5% of the variance in vaccine acceptance. Results of the multivariate logistic regression analysis are shown in Table 6. The Pearson goodness-of-fit test yielded a χ^2^_1474_ of 1500.56, which was considered insignificant (*P*=.31). The deviance goodness-of-fit test yielded a χ^2^_1474_ of 1374.86, which was also considered insignificant (*P*=.97). These results suggest good model fit.

**Table 6:** Multivariate logistic regression analysis of vaccine acceptance among individuals with serious comorbidities (n=21,294) using Inspire between January 15, 2021 and February 22, 2021.

Variable		Odds ratio (95% CI)	*P* value
Routine influenza vaccine		1.08 (1.07-1.08)	<.001
Trust in responsible development of COVID vaccine		1.86 (1.84-1.88)	<.001
Prior COVID infection		0.97 (0.96-0.98)	<.001
**Political leaning**			
	Independent	1.02 (1.01-1.03)	<.001
	Liberal	1.06 (1.05-1.07)	<.001
Residence (United States vs outside the United States)		0.98 (0.98-0.99)	<.001
**Age**			
	Median age	1.01 (0.99-1.02)	.07
	Above the median age	1.02 (1.01-1.03)	<.001
**Education level**			
	Some college	1.00 (0.99-1.01)	.56
	College and graduate school	0.99 (0.98-1.01)	.68
Smoking status		1.01 (1.00-1.02)	.004
Gender		1.00 (0.99-1.02)	.67
Cancer diagnosis		1.00 (0.99-1.00)	.45
Mask wearing		0.10 (0.99-1.01)	.96

Factors associated with vaccine acceptance after controlling for other covariates included routine influenza vaccination, political leaning, age (below the median versus median range versus above the median), country of residence (in the United States versus living outside the United States), prior COVID-19 infection, and trust in responsible development of the COVID-19 vaccine. Routine receipt of influenza vaccination remained a positive predictor of COVID-19 vaccine acceptance after controlling for other covariates, with an OR of 1.08, meaning participants who reported regularly receiving the flu shot had 1.08 times the odds of being vaccine acceptant. Trust in responsible development of the vaccine was also a significant predictor of COVID-19 vaccine acceptance, with an OR of 1.86, meaning that those who reported having trust in the development of the vaccine had 1.86 times the odds of receiving it than those that reported not having trust in the development. Those residing in the United States (OR 0.98) had 0.98 times the odds of accepting the vaccine than those living outside the United States. Those who reported never smoking also had slightly greater odds of vaccine acceptance (OR 1.01). By contrast, vaccine acceptance was less likely with a history of prior COVID-19 infection (OR 0.97). After controlling for other variables, those reporting an independent political leaning had 1.02 times the odds of being vaccine acceptant compared with those who reported a conservative political leaning, and those who reported a liberal political leaning had 1.02 times the odds of being vaccine acceptant than those who reported a conservative political leaning. Age remained a statistically significant predictor of vaccine acceptance after controlling for other variables. Those with an age higher than the median age of the study had 1.02 times the odds of reporting vaccine acceptance compared with those below the median age, while those at the median age had 1.01 times the odds of being vaccine acceptant compared with those below the median age. When controlling for other variables, gender was no longer a statistically significant predictor of vaccine acceptance. The same is true for education level, cancer diagnosis, and mask wearing.

### Concerns About Vaccines

Of the 3960 respondents who indicated COVID-19 vaccine hesitancy, apprehension regarding the newness of the vaccine was the most commonly cited reason for hesitancy, expressed by 53.1% (2104/3960) of hesitant respondents. Concerns about the safety of the vaccine and a general distrust of the development process (including governmental oversight) also were common (Table 7).

**Table 7:** Concerns about the COVID-19 vaccine among the vaccine-hesitant individuals (n=3960) using Inspire between January 15, 2021 and February 22, 2021.

Responses to the question: “What are your concerns about the vaccine? Check all that apply.”	Overall (n=3960), n (%)	United States (n=2817), n (%)	Outside the United States (n=1143), n (%)
I am concerned the COVD-19 vaccine is too new.	2104 (53.1)	1532 (54.4)	572 (50.0)
I do not trust the government has ensured that the vaccines are safe and effective.	1900 (48.0)	1365 (48.5)	535 (46.8)
I am concerned about side effects and discomfort.	1738 (43.9)	1219 (43.4)	519 (45.5)
I do not trust the COVID-19 vaccine in particular.	1571 (39.7)	1126 (40.0)	445 (38.9)
I have concerns over the role of politics in the development process.	1533 (38.7)	1112 (39.4)	421 (36.8)
I want to see how others respond first.	1319 (33.3)	974 (34.6)	345 (30.2)
I do not think it was developed responsibly.	1313 (33.2)	922 (32.7)	391 (34.2)
I do not believe I need it.	869 (22.0)	589 (20.9)	280 (24.5)
I do not trust vaccines in general.	832 (21.0)	591 (21.0)	292 (25.5)
I have religious objections.	331 (8.4)	262 (9.3)	69 (6.0)
I am concerned with contracting the coronavirus from the vaccine.	327 (8.3)	221 (7.8)	106 (9.3)
It is too difficult to get vaccinated.	86 (2.2)	74 (2.6)	12 (1.0)

### Early Experience With COVID-19 Vaccination in High-Risk Populations

**Figure 1:**
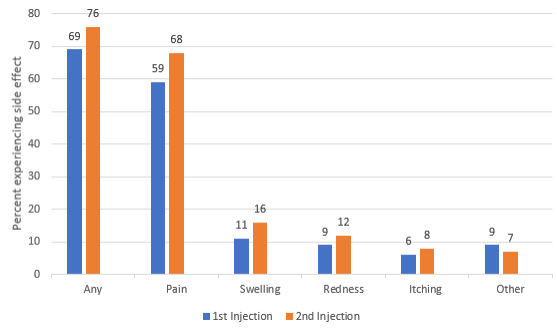
Self-reported localized reactions to COVID-19 vaccination among individuals with cancer, autoimmune diseases, or other serious comorbidities and/or their caregivers (n=5501 who received an initial vaccine dose; n=1390 who completed a 2-dose series).

**Figure 2:**
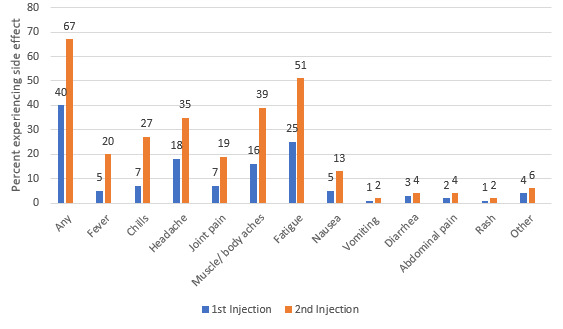
Self-reported systemic reactions to COVID-19 vaccination among individuals with cancer, autoimmune diseases, or other serious comorbidities and/or their caregivers (n=5501 who received an initial vaccine dose; n=1390 who completed a 2-dose series).

As of the study cutoff, 5501 (5501/21,294, 25.8%) survey respondents had received at least one COVID-19 vaccination (Pfizer-BioNTech: 2640/5501, 48.0%; Moderna: 2586/5501, 47.0%; Oxford-AstraZeneca: 55/5501, 1.0%; other/unknown: 220/5501, 4.0%). A 2-injection series was completed by 6.5% (1390/21,294) of respondents. Following the first injection, 69.0% (3796/5501) self-reported experiencing local adverse events, and 40.0% (2200/5501) self-reported systemic reactions. Pain at the injection site was the most commonly self-reported side effect. Fatigue and myalgias were the most commonly self-reported systemic side effects. Among those who had received 2 vaccine injections (n=1390), the frequencies of self-reported local and systemic reactions increased following the second injection, to 77.0% (1070/1390) and 67.0% (931/1390), respectively (Figures 1 and 2).

Among respondents who had received a vaccination with the Pfizer-BioNTech (n=2640) or Moderna (n=2586) vaccines, the initial injection led to overall self-reported localized side effects among 65.0% (1716/2640) and 75.0% (1939/2586), respectively. Local reactions increased to 72.0% (480/667) and 85.0% (368/433) with the second booster Pfizer-BioNTech and Moderna injections, respectively. A more dramatic increase in self-reported systemic side effects was noted with the second injection, with overall systemic effects rising from 37.0% (977/2640) to 62.0% (413/667) and 40.0% (1034/2586) to 77.0% (333/433), with the Pfizer-BioNTech and Moderna vaccines, respectively.

Of the 5459 cancer patients who responded to the survey, 30.0% (1638/5459) had received 1 injection, and 6.0% (325/5459) completed both vaccine injections*. *In this cancer population, 64.5% (1057/1638) self-reported local reactions, and 34.1% (559/1638) self-reported systemic reactions to the first injection; with the second injection, 72.3% (235/325) experienced local reactions, and 59.1% (192/325) experienced systemic reactions. The types of reactions mirrored the overall study population*. *Of the 5186 individuals with autoimmune disorders, 23.9% (1239/5186) had received 1 vaccination, and 6.0% (311/5186) had completed the series. In this immunocompromised population, with the first injection, local reactions were described by 69.0% (855/1239), and systemic reactions were described by 41.3% (512/1239); with the second vaccine injection, local reactions were described by 78.1% (243/311), and systemic reactions were described by 67.2% (209/311). Among the 1878 respondents with chronic lung diseases who received the vaccine, with the first injection, 67.2% (1262/1878) self-reported local reactions, and 39.9% (794/1878) self-reported systemic reactions; with the second vaccine injection, local reactions occurred in 76.8% (288/375), and systemic reactions occurred in 69.1% (259/375). Similar patterns were noted among respondents with obesity (1st dose: 539/777, 69.4% had local reactions, and 334/777, 43.0% had systemic reactions; 2nd dose: 154/202, 76.2% had local reactions, and 152/202, 75.2% had systemic reactions), hypertension (1st dose: 947/1420, 66.7% had local reactions, and 550/1420, 38.7% had systemic reactions; 2nd dose: 272/366, 74.3% had local reactions, and 243/366, 66.5% had systemic reactions), and type 2 diabetes (1st dose: 253/376, 67.3% had local reactions, and 159/376, 42.3% had systemic reactions; 2nd dose: 74/95, 77.9% had local reactions, and 75/95, 78.9% had systemic reactions). 

## Discussion

In this survey of nearly 22,000 individuals with serious comorbid conditions conducted shortly after vaccine regulatory approvals, 8 in 10 respondents reported a willingness to receive the COVID-19 vaccine. This high level of vaccine acceptance in a community of vulnerable individuals who regularly seeks medical information through participation in an online health forum compares favorably with reports in public opinion polls drawn from general populations taken at the same timeframe [6,7]. Additionally, as of late February 2021, 29.6% (4813/16,277) of US participants in the survey stated that they had already received at least one COVID-19 vaccine injection, which compared favorably with the 18% vaccination prevalence in US adults at that time [24]. Our survey thus appears to confirm a strong desire for protection against SARS-CoV-2 in vulnerable populations, although vaccine allocation prioritization may have also influenced these findings.

However, almost 1 in 5 respondents to our survey, all of whom had comorbidities, reported COVID-19 vaccine hesitancy. This was a similar hesitancy prevalence as reported in general population polls at the time [6,7]. Among patients with cancer, autoimmune diseases, and chronic lung diseases, 13.4%, 19.4%, and 17.8%, respectively, expressed hesitancy. This is very concerning given that individuals with cancer and other serious comorbidities have experienced an increased proportion of the mortality from the pandemic [13-18]. Furthermore, since our survey enrolled from a medically savvy population who participate in online health forums, we were surprised by these results. The lack of inclusion of immunocompromised individuals within regulatory clinical trials may have contributed to the safety concerns expressed by 43.9% of vaccine-hesitant respondents [20,21]. However, other factors, many of which were similar to concerns raised by the general public, were deemed important by our respondents. Thus, it appears that our study population fell into 2 polarizing cohorts: one group that was more eager to undergo vaccination as a consequence of coexisting illnesses and increased mortality risks and a second group that was COVID-19 vaccine hesitant and influenced by broad social vaccine concerns.

We identified multiple factors that were independently associated with vaccine hesitancy. Lack of trust in COVID-19 vaccine development, including the rapidity and politicization of the process, was expressed by our comorbid cohort but is a view not unique to our population [12]. Generalized distrust of vaccines and avoidance of influenza vaccines were additional broad concerns that transcend comorbid status. Conservative political leaning, lower education level, and younger age are also commonly cited in public opinion polls [7,8,10,11,25]. Individuals who had already contracted COVID-19 avoided vaccination, possibly believing natural immunity alone was protective [26].

Few studies have specifically explored issues of COVID-19 vaccine hesitancy among patients with severe comorbid conditions or strategies to increase acceptance in high-risk populations. As these individuals already have ongoing health care contact, the potential influence of their physicians should not be ignored. A Korean study noted that, although only 61.8% of their cancer patients were initially willing to receive the COVID-19 vaccine, acceptance increased by 30% if their oncologist recommended it [27]. Similarly, a Tunisian study noted that a discussion about the impact of COVID-19 upon cancer treatments and outcomes was projected to have the single greatest impact on reducing hesitancy [28]. An online survey of 540 Mexican women with breast cancer also noted a 3-fold increase in the likelihood of accepting vaccination following their oncologists’ recommendation [29]. Unfortunately, a physician’s recommendation does not always change opinions. Nearly 40% of French cancer patients who were vaccine hesitant did not feel that their oncologist was qualified to advise them on COVID-19 vaccination and instead preferred to rely on personal judgements [30]. Nonetheless, the specialist physician possesses unique insights into potential impacts of vaccination on the patient’s underlying disease, a fear that must be allayed, as expressed by a cohort of patients with autoimmune rheumatic disease [31]. A UK randomized trial demonstrated that emphasizing the personal benefits of vaccination reduced hesitancy to a greater extent than information about collective benefit. Where perception of risk from vaccines is most salient, which is likely among high-risk comorbid populations, decision making frequently becomes centered on the personal [32].

Establishing trust in science and vaccine development is critical to reducing vaccine hesitancy. Despite our population having ongoing contact with the health care system (by virtue of their underlying diseases) and routinely engaging in an online health-related forum, we noted that issues regarding trust were expressed by over 40% of vaccine-hesitant respondents. A survey of nearly 6000 US health care workers, older adults, frontline essential workers, other essential workers, and individuals with a high-risk chronic condition conducted in early 2021 identified that lack of trust in the vaccine approval and development processes was the most important trust issue. Other domains of trust (in vaccine safety and efficacy, in health care providers, in sources of information, and generalized trust) were of lesser importance [33]. Similar results were noted in an online survey of over 1000 Italians who responded that vaccine acceptance was driven by a trust in science, acceptance of prior vaccines, and an understanding that COVID-19 is more serious than influenza [34].

The potential role of social media in combating the COVID-19 pandemic cannot be underestimated. This study was sponsored by an online health community whose international membership shares medical information and personal experiences via hundreds of disease-specific forums. Our motivation for designing the study was to increase our membership’s knowledge and encourage discussions regarding COVID-19 vaccine experiences. The rapid enrollment of nearly 22,000 respondents with serious diseases over a 5-week period, with thousands more viewing the online results, attests to the potential influence of the worldwide web on health issues. An infodemiology study of over 650,000 “tweets” from November 2020, prior to the release of vaccines, identified that the main themes driving vaccine hesitancy were concerns of safety, efficacy, freedom, and mistrust in institutions (either the government or multinational corporations) [35]. A qualitative coding methodologic review of antivaccine social media noted that the most frequent narratives centered on “corrupt elites” and rhetoric appealing to the vulnerability of children [36]. As rumors and conspiracy theories are common, tracking COVID-19 vaccine misinformation in real time and engaging with social media to disseminate correct information can be an important safeguard against misinformation [37]. Health care–related patient platforms, such as Inspire, where individuals with concerns can obtain understandable COVID-19–related medical information relevant to their other medical conditions should play an important role in decreasing vaccine hesitancy.

As noted in our survey, COVID-19 vaccine acceptance and hesitancy are a global issue. Respondents residing outside the United States were more likely to exhibit vaccine hesitancy, but the reasons for concerns about vaccination appeared similar. A systematic review of World Health Organization regions noted great variability in acceptance of the vaccine, with lowest rates in Hong Kong and the Democratic Republic of the Congo, 2 countries with recent political instability. In contrast, China, Indonesia, and Malaysia all reported hesitancy prevalence below 10%, potentially a reflection of their early experiences with SARS-CoV-2. Across Europe, hesitancy varied greatly from 20% in the United Kingdom to almost 60% in Italy [38]. Other reports have indicated higher acceptance of vaccination in lower- and middle-income countries [39,40]. As evidenced by the 123 nations represented in our respondent population, the internet represents a powerful potential tool for dissemination of information about COVID-19 vaccination across boundaries.

Limited data exist regarding the safety and effectiveness of COVID-19 vaccination among immunocompromised individuals (with the exception of individuals infected with HIV) since they were excluded from the regulatory phase 3 trials. Therefore, we expected safety concerns to dominate vaccine hesitancy concerns in our survey [41]. To address this, we requested information about side effect profiles among respondents who had undergone vaccination with the goal of sharing this information with our online membership in the hope that this would reduce vaccine hesitancy. Indeed, early experience with vaccinations, as self-reported by the over 5000 respondents who had already been vaccinated, should be reassuring to individuals with serious comorbidities. Side effect profiles were similar to adverse event reports from the regulatory trials, although overall generally lower in frequency [23,42]. Whether this is a reflection of the weaker immune status of our population or a result of differences in reporting styles (online survey vs research-grade clinical trial monitoring) is unknown. However, an interesting finding was that the prevalence of self-reported systemic reactions to the initial vaccination appeared to be much lower than those reported in the clinical regulatory trials but increased, closer to the general population results, with the booster. This pattern of side effect intensity (as a surrogate for immune responsiveness) suggests that booster vaccines may be required in immunocompromised individuals or that confirmation of antibody response may be necessary. Regardless, given the side effect profiles noted in our survey, the recommendations to vaccinate individuals with potential immune dysfunction despite a lack of clinical trial data appear justified, although future studies to document vaccine efficacy in these populations are needed.

We recognize several limitations to our study. The survey was conducted in January 2021 and February 2021, shortly after the release of the COVID-19 vaccine, and represents attitudes from a single time point. As additional information about the safety and efficacy of vaccination becomes available to our participants, we expect that attitudes might change. Indeed, serial tracking polls conducted by the Kaiser Family Foundation have noted an increase in the acceptance of vaccination over time, although most of the changes in attitudes have occurred among the “wait and see” populations, with little movement among the vaccine-hesitant cohort [8]. Nonetheless, it is probable that our findings do not represent current opinions. Additionally, ORs determined by logistic regression analysis do not approximate relative risk or prevalence ratios since the outcome variable of vaccine hesitancy was not rare in our study population [43]. Additionally, the Inspire community membership is 77% female with a median age of 40-49 years. Given the composition of Inspire’s community, survey respondents were not intended to represent a random sampling of the general population or any outside demographic. We also obtained a low (2.2%) response rate to our online survey, and thus, our findings might not be representative of our membership population. It is possible that the most vocal opinions were overexpressed. We noted a dichotomous response, with a larger cohort desiring vaccination (more than the general population) but also a significant vaccine-hesitant cohort, with few respondents in the middle. It is interesting that our vaccine hesitancy prevalence and concerns mirror those of general population opinion polls, indicating that vulnerable populations are susceptible to antivaccination social issues. Additionally, although we noted several factors that appeared to be associated with vaccine hesitancy or acceptance, a cause-and-effect relationship should not be inferred on the basis of our survey. Finally, we did not investigate methods to reduce vaccine hesitancy in this study but plan to add items to ongoing online surveys of our membership with this goal.

In summary, our online survey highlights a high level of acceptance of COVID-19 vaccines among vulnerable individuals. However, the finding that 1 in 5 remains vaccine hesitant is of concern and points to a need for additional efforts. Although governmental mandates or financial incentives are being considered, educational efforts must continue [44,45]. Among individuals who have serious comorbid diseases and thus are already connected to the health care system, direct conversations by the medical specialist team about the impact of the COVID-19 vaccine have been demonstrated to reduce hesitancy and should be intensified. As demonstrated by our survey, it cannot be assumed by physicians that the most medically vulnerable automatically accept vaccination. Disinformation about the COVID-19 vaccines is common on social media sites and fosters hesitancy [46]. Our intent is to share our study results with the ≥2 million members of the Inspire health community, harnessing the internet to increase vaccine acceptance by demonstrating tolerable vaccine side effects among individuals with serious comorbid conditions. A website detailing the survey questions and updated daily with results is available to the general public [47].

## Abbreviations

OR: odds ratio
